# Antimicrobial Furoquinoline Alkaloids from *Vepris* *lecomteana* (Pierre) Cheek & T. Heller (Rutaceae)

**DOI:** 10.3390/molecules23010013

**Published:** 2017-12-21

**Authors:** Ariane Dolly Kenmogne Kouam, Achille Nouga Bissoue, Alain Tadjong Tcho, Emmanuel Ngeufa Happi, Alain François Kamdem Waffo, Norbert Sewald, Jean Duplex Wansi

**Affiliations:** 1 Department of Chemistry, University of Douala, Faculty of Sciences, 24157 Douala, Cameroon; arydolls@gmail.com (A.D.K.K.); anbissoue@yahoo.fr (A.N.B.); ngeufa@yahoo.fr (E.N.H.); akamdemfr@yahoo.fr (A.F.K.W.); 2 Organic and Bioorganic Chemistry, Department of Chemistry, Bielefeld University, 33501 Bielefeld, Germany; 3 Department of Chemistry, University of Buea, Faculty of Sciences, 63 Buea, Cameroon; alainstone1@yahoo.fr

**Keywords:** *Vepris lecomteana*, furoquinoline alkaloids, lecomte quinoline A–C

## Abstract

Three new prenylated furoquinoline alkaloids named lecomtequinoline A (**1**), B (**2**), and C (**3**), together with the known compounds anhydroevoxine (**4**), evoxine (**5**), dictamnine (**6**), *N-*methylflindersine (**7**), evoxanthine (**8**), hesperidin, lupeol, *β*-sitosterol, stigmasterol, *β*-sitosterol-3-*O*-*β*-d-glucopyranoside, stearic acid, and myristyl alcohol, were isolated by bioassay-guided fractionation of the methanolic extracts of leaves and stem of *Vepris lecomteana*. The structures of compounds were determined by spectroscopic methods (NMR, MS, UV, and IR) and by comparison with previously reported data. Crude extracts of leaves and stem displayed high antimicrobial activity, with Minimum Inhibitory Concentration (MIC) (values of 10.1–16.5 and 10.2–20.5 µg/mL, respectively, against *Escherichia coli*, *Bacillus subtilis*, *Pseudomonas agarici*, *Micrococcus luteus*, and *Staphylococcus warneri*, while compounds **1**–**6** showed values ranging from 11.1 to 18.7 µg/mL or were inactive, suggesting synergistic effect. The extracts may find application in crude drug preparations in Western Africa where *Vepris lecomteana* is endemic, subject to negative toxicity results in vivo*.*

## 1. Introduction

*Vepris lecomteana* (Pierre) Cheek & T. Heller (Rutaceae), previously called *Oricia lecomteana* Pierre, is an evergreen shrub up to 15 to 20 m tall with alternate leaves and three foliolates rounded at the base. The species is widespread in dense and humid forests of Nigeria, Cameroon, Gabon, and Congo [1]. Species of the genus *Vepris* are employed ethnomedicinally in the treatment of a diverse range of ailments, including pneumonia, lung diseases and kidney disorders, ocular diseases, cardiac pains, coughs, colds and influenza, headache, menorrhagia and infertility, as an aphrodisiac, diuretic, and antipyretic, astringent and fortifier, tonic for angina and rheumatism, and both orally and externally as a treatment for malaria [2]. Several secondary metabolites, such as acridones, furoquinolines, quinolones, amides, azoles, coumarins, fatty acids, flavonoids, indoloquinazolines, limonoids, lignans, phenolic compounds, and terpenoids, have been reported from *Vepris* species [2,3,4,5,6,7,8,9,10,11,12,13]. Some of these compounds exhibit potent anti-inflammatory, antibacterial, antioxidant, antimalarial, and cytotoxic activity [8,9,10,11,12,13,14]. This study represents the first report on phytochemical and pharmacological properties of *V. lecomteana*, which is used in traditional medicine against bacterial infections, and, thus, the potential of this work is as research aimed at discovering new and infective agents.

## 2. Results and Discussion

**Figure 1:**
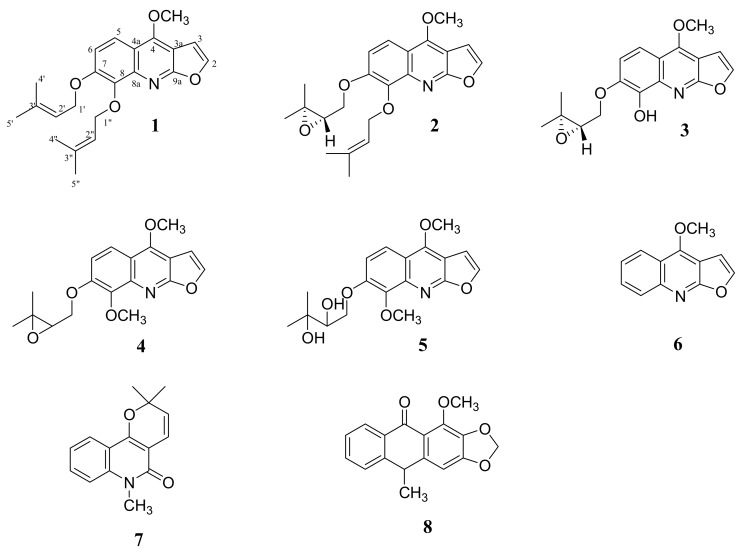
Structures of some isolated compounds.

Leaves and stem of *Vepris lecomteana* were extracted separately with MeOH. The methanolic extracts were fractionated using vacuum liquid chromatography (VLC). Successive purifications by column chromatography and preparative thin layer chromatographic (pTLC) afforded three new furoquinoline alkaloids (**1**–**3**), together with twelve known compounds. By comparison with previous data, the known compounds were identified as anhydroevoxine (**4**), evoxine (**5**), dictamnine (**6**), *N*-methylflindersine (**7**), evoxanthine (**8**), hesperidin, lupeol, *β*-sitosterol, stigmasterol, *β*-sitosterol-3-*O*-*β*-d-glucopyranoside, stearic acid, and myristyl alcohol [15,16,17,18] (Figure 1).

Compound **1** was isolated as white amorphous needles. The molecular formula C_22_H_25_NO_4_ was deduced from the HR-ESI-MS ([M + Na]^+^ at *m*/*z* 390.1730, calcd. 390.1783). The UV absorption bands at 229, 307, and 339 nm, and the IR spectrum (3180, 3030, 2974, 2929, 1616, 1574, and 1363 cm^−1^) indicated **1** to be a furoquinoline alkaloid [19]. The ^13^C-NMR spectrum of the compound in combination with a Distortionless Enhancement by Polarisation Transfer (DEPT) spectrum exhibited a total of 22 carbon resonances, attributed to five methyl, two methylene, six methine, and nine quaternary carbons (Table 1). The ^1^H-NMR spectrum of **1** displayed a lower field methoxy group at *δ_H_* 4.35 of the 4-methoxyfuroquinoline alkaloid group [19], with a pair of coupled doublet resonances at *δ_H_* 7.50 and 6.96 (each 1H, *J* = 2.8 Hz), characteristic of the H-2 and H-3 of furan ring protons and two coupled doublet protons resonance at *δ_H_* 7.13 and 7.90 (each 1H, *J* = 9.3 Hz), attributed to aromatic protons H-5 and H-6. In addition, the ^1^H-NMR spectrum contained signals corresponding to two olefinic protons at *δ_H_* 5.49 (m) and 5.67 (m), two oxymethylenes at *δ_H_* 4.75 (d, *J* = 7.1 Hz, H-1′) and 4.68 (d, *J* = 6.7 Hz, H-1″), and four methyls linked to vinylic carbons, each appearing as singlet at *δ_H_* 1.59; 1.66; 1.69; 1.71 attributable to two prenyloxy side chain substituents attached to the skeleton. The complete assignment of compound **1** was based on Correlation Spectroscopy (COSY), Heteronuclear Multiple Quantum Connectivity (HMQC), and Heteronuclear Multiple Bond Connectivity (HMBC) experiments. In the HMBC spectrum, correlations between H-5 (*δ_H_* 7.90), C-4 (*δ_C_* 157.1), C-8a (*δ_C_* 142.1), and C-7 (*δ_C_* 151.9); H-6 (*δ_H_* 7.13) and C-4a (*δ_C_* 114.9), C-8 (*δ_C_* 141.8), and C-7 (*δ_C_* 151.9), as well as between H-1′ (*δ_H_* 4.75) and C-7 (*δ_C_* 151.9), C-3′(*δ_C_* 137.4), C-4′(*δ_C_* 25.8), and C-5′ (*δ_C_* 18.0); H-1″ (*δ_H_* 4.68) and C-8 (*δ_C_* 141.8); C-3″ (*δ_C_* 137.6), C-4″(*δ_C_* 25.8) and C-5″ (*δ_C_* 18.3) indicated that the two prenyloxy substituents are attached to position C-7 and C-8. The orientation of the furan ring was precisely determined by 2D-NMR techniques HMBC and Nuclear Overhauser Effect Spectroscopy (NOESY). In the HMBC spectrum, furan proton H-2 (*δ_H_* 7.50) showed correlations with the carbon signals at C-9a (*δ_C_* 164.1) and C-3a (*δ_C_* 101.9), and the H-3 (*δ_H_* 6.96) furan proton showed correlations with the carbon signals at C-9a (*δ_C_* 164.1), C-4 (*δ_C_* 157.1), C-3a (*δ_C_* 101.9), and OCH_3_-4 (*δ_C_* 58.9). Furthermore, in the NOESY spectrum, the cross peaks observed between furan proton H-3 (*δ_H_* 6.96) and OCH_3_-4 (*δ_H_* 4.35), and between the OCH_3_-4 (*δ_H_* 4.35) and proton H-5 (*δ_H_* 7.90), clearly indicated that the furan ring is fused to the quinoline nucleus at the position [2,3-*b*].

From the above spectroscopic data, the structure of compound **1** was determined as 4-methoxy-7,8-*bis*(3-methylbut-2-enyloxy)furo[2,3-*b*]quinoline, and was named lecomtequinoline A.

Compound **2** was obtained as white needles. The molecular composition was found to be C_22_H_25_NO_5_ by HR-ESI-MS ([M]^+^ at *m*/*z* 383.17280, calcd. 383.17327). This value was 16 mass units higher than that of compound **1**, suggesting the presence of one additional oxygen in compound **2**. The UV absorption bands (228, 252, 310 and 316 nm) and the IR spectrum (3118, 2969, 2860, 1674, 1576, 1360 and 1290 cm^−1^) indicated a 4-methoxyfuroquinoline alkaloid skeleton for compound **2** as well **[19]**. The ^1^H-NMR, ^13^C-NMR, DEPT, COSY, HMQC and HMBC spectra showed the presence of the same aromatic spin systems as in compound **1** (Table 1). The ^1^H-NMR spectrum revealed the presence of one prenyloxy (*δ_H_* 5.67, 4.77, 1.67, and 1.61) substituent attached to C-8 and the 2,3-epoxyprenyloxy group at *δ_H_* 4.32 (dd, *J* = 11.3; 4.9 Hz, H-1′a), 4.21 (dd, *J* = 11.3; 5.6 Hz, H-1′b), 3.16 (dd, *J* = 5.6; 4.9 Hz, H-2′), 1.32 (d, *J* = 1.5 Hz, H-4′) and 1.28 (d, *J* = 1.3 Hz, H-5′). These values show that the double bond of the prenyloxy group was oxidized to give an epoxide. The ^13^C-NMR and DEPT spectra confirmed the presence of the 2,3-epoxyprenyloxy group at *δ_C_* 69.4, 61.6, 58.3, 24.6 and 18.9 (Table 1). The position of this group at C-7 was confirmed by correlations observed in the HMBC spectrum between H-1′ (*δ_H_* 4.32) and C-7 (*δ_C_* 151.6), C-3′ (*δ_C_* 58.3), C-4′ (*δ_C_* 24.6) and C-5′ (*δ_C_* 18.9). The orientation of the furan ring was precisely determined by 2D-NMR techniques HMBC and NOESY. In the HMBC spectrum, furan proton H-2 (*δ_H_* 7.51) showed correlations with the carbon signals at C-9a (*δ_C_* 164.2) and C-3a (*δ_C_* 102.2) and the H-3 (*δ_H_* 6.96) furan proton, showed correlations with the carbon signals at C-9a (*δ_C_* 164.2), C-4 (*δ_C_* 157.1), C-3a (*δ_C_* 102.2) and OCH_3_-4 (*δ_C_* 59.0). Furthermore, in the NOESY spectrum, the cross peaks observed between furan proton H-3 (*δ_H_* 6.96) and OCH_3_-4 (*δ_H_* 4.35), and between the OCH_3_-4 (*δ_H_* 4.35) and proton H-5 (*δ_H_* 7.91), clearly indicated that the furan ring is fused to the quinoline nucleus at the position [2,3-*b*]. The absolute configuration of **2** was determined on the basis of circular dichroism (CD) spectroscopic analysis. Thus, the CD spectrum of **2** showed a positive Cotton effect [250 (Δε +4.82), 242 (Δε +0.70) nm] in the same region as (*S*)-nkolbisine [20], which indicates the absolute configuration at C-2′ to be *S*. From above spectroscopic studies, the structure of compound **2** was determined as (*S*)-(−)-7- [(2,3-epoxy-3-methylbutyl)oxy]-8-(3-methylbut-2-enyloxy)-4-methoxyfuro[2,3-*b*]quinoline and named lecomtequinoline B.

Compound **3** was obtained as a white powder. The molecular composition was found to be C_17_H_17_NO_5_ by HR-ESI-MS ([M]^+^ at *m*/*z* 315.1087, calcd. 315.1106). This value was 68 mass units lower than that of compound **2**, suggesting the absence of the prenyl group (C_5_H_8_) in compound **3**. According to its UV spectrum (232, 255, 305, and 325 nm) and IR (3224, 3126, 2971, 1625, 1584, 1378, and 1240 cm^−1^) which showed the same characteristic values as in **2**, compound **3** belongs to the furoquinoline alkaloid group as well. The absence of the prenyl group was confirmed by the ^1^H and ^13^C-NMR spectra (Table 1). The ^1^H-NMR showed the presence of the 2,3-epoxyprenyloxy moiety at *δ_H_* 4.44 (dd, *J* = 11.1; 5.0 Hz, H-1′a), 4.04 (dd, *J* = 11.1; 6.0 Hz, H-1′b), 3.18 (dd, *J* = 6.0; 5.0 Hz, H-2′), 1.37 (d, *J* = 1.3 Hz, H-4′), and 1.36 (d, *J* = 1.3 Hz, H-5′), the free hydroxyl group at *δ_H_* 4.55 (brs), exchangeable with D_2_O. The ^13^C-NMR spectrum exhibited only 17 carbons compared to 22, as for compound **2**. In the HMBC spectrum, correlations observed between H-1′ (*δ_H_* 4.44) and C-7 (*δ_C_* 142.6), C-3′ (*δ_C_* 58.7), C-4′ (*δ_C_* 24.2), and C-5′ (*δ_C_* 23.5) indicate that the 2,3-epoxyprenyloxy substituent is attached to position C-7, and the free hydroxyl is present at C-8. The orientation of the furan ring was precisely determined by 2D-NMR techniques HMBC and NOESY. In the HMBC spectrum, furan proton H-2 (*δ_H_* 7.74) showed correlations with the carbon signals at C-9a (*δ_C_* 163.8) and C-3a (*δ_C_* 101.9), and the H-3 (*δ_H_* 7.26) furan proton showed correlations with the carbon signals at C-9a (*δ_C_* 163.8), C-4 (*δ_C_* 157.7), C-3a (*δ_C_* 101.9), and OCH_3_-4 (*δ_C_* 58.6). Furthermore, in the NOESY spectrum, the cross peaks observed between furan proton H-3 (*δ_H_* 7.26) and OCH_3_-4 (*δ_H_* 4.43), and between the OCH_3_-4 (*δ_H_* 4.43) and proton H-5 (*δ_H_* 7.65), clearly indicated that the furan ring is fused to the quinoline nucleus at the position [2,3-*b*]. Thus, the CD spectrum of **3** showed the same positive Cotton effect [276 (Δε +4.92), 232 (Δε +0.90) nm] in the same region as (*S*)-nkolbisine [20], which indicates the *S*-configuration at C-2′. From above data, compound **3** was characterized as (*S*)-(−)-7-(2,3-epoxy-3-methylbutyloxy)-4-methoxyfuro[2,3-*b*]quinolin-8-ol, and named lecomte quinoline C.

Since species of *Vepris* are used in traditional medicine for the treatment of bacterial infections related to forms of pneumonia, ocular diseases, cardiac pains, coughs, colds, angina, and fever [2], antibacterial properties of leaves and stem crude extracts, fractions, and some of the isolated compounds, were investigated. The microdilution assay gave high activities for the methanolic extract of leaves and stem, with MIC values of 10.1–16.5 and 10.2–20.5 µg/mL, respectively. While fraction C of the leaf extract showed enhanced activity against *Micrococcus luteus* with MIC = 4.5 µg/mL, fraction C′ of the stem extract showed good activities, with MIC values against *Escherichia coli*, *Micrococcus luteus*, and *Staphylococcus warneri* of 10.5, 10.7, and 13.5 µg/mL, respectively. Lecomtequinoline A-C (**1**–**3**) and anhydroevoxine (**4**) isolated from the leaf extract, and evoxine (**5**) and dictamnine (**6**) from the stem extract, however, gave slightly lower activities, with MIC values ranging from 11.1 to 18.7 µg/mL, or were inactive (Table 2). Previously, the water-soluble alkaloid fraction of *Vepris louisii* occurring in Western Africa had shown significant antibacterial activity, and delivered the dihydrofuroquinoline alkaloid veprisinium chlorid exhibiting broad and high activity against a number of clinical bacterial isolates [4]. In the following, *Vepris lanceolata*, endemic to Mauritius, delivered from its hexane and methanol/chloroform fractions of the stem MICs of 32 and 16 mg/mL, respectively, against *Pseudomonas aeruginosa* as well as against *S. aureus*, which is around 1000-fold less active than MICs received against human pathogen strains reported here from *Vepris lecomteana*. In addition, the plant’s methanol/chloroform fraction from leaves was not active against *Pseudomonas aeruginosa* at all, and displayed a very low MIC of 16 mg/mL against *Staphylococcus aureus* [21]. Furthermore, flindersine isolated from the chloroform/methanol extract of the wood of *Vepris punctata*, occurring in the Madagascar rain forest, was later reported, as well, from the ethyl acetate extract of the leaves of another medicinal plant belonging to the Rutaceae family and tested against *Bacillus subtilis*, *Staphylococcus aureus*, *Staphylococcus epidermidis*, *Enterococcus faecalis*, *Pseudomonas aeruginosa*, and *Acinetobacter baumannii*, delivering low MICs of 31.25, 62.5, 62.5, 31.25, 250, and 125 µg/mL, respectively [22,23]. Interestingly, antibacterial testing of an epoxidized prenylated cinnamaldehdye derivative from *Vepris glomerata,* East Africa*-*named glomeral, provided significant MICs of 2 µg/mL and 0.4 µg/mL against standard strains of *Staphylococcus aureus* and *Salmonella dysentrieae,* respectively, giving a rationale for the use of this plant in the treatment of bacterial infections [12]. It should be noted that *Vepris* species are widely used in traditional African medicine against multiple diseases, including various bacterial infections, probably indicating a medically valuable metabolite spectrum still to be detected from this genus.

## 3. Materials and Methods

### 3.1. General 

Optical rotation indices were determined in methanol on a JASCO DIP-3600 digital polarimeter (JASCO, Tokyo, Japan) using a 10 cm cell. CD spectra were measured on a JASCO J-810 spectropolarimeter (JASCO). IR spectra were determined on a JASCO Fourier transform IR-420 spectrometer (JASCO). Ultraviolet spectra were recorded on a Hitachi UV 3200 spectrophotometer in MeOH and infrared spectra on a JASCO 302-A spectrophotometer (Thermo Scientific, Waltham, MA, USA). ESI-HR mass spectra were measured on Agilent Techn. 6220 TOF LCMS mass spectrometer (Agilent Technologies, Santa Clara, CA, USA) and EI-MS on a Finnigan MAT 95 spectrometer (70 ev) (Thermo Fischer Scientific, Darmstadt, Germany) with perfluorokerosene as reference substance for ESI-HR-MS. The ^1^H- and ^13^C-NMR spectra were recorded at 500 MHz and 125 MHz, respectively, on Bruker DRX 500 NMR spectrometers (Bruker Corporation, Brussels, Belgium) in CDCl_3_. Methyl, methylene, and methine carbons were distinguished by DEPT experiments. Homonuclear ^1^H connectivities were determined by using the COSY experiment. One-bond ^1^H-^13^C connectivities were determined with HMQC gradient pulse factor selection, and two- and three-bond ^1^H-^13^C connectivities by HMBC experiments. Chemical shifts are reported in *δ* (ppm) using Tetramethylsilane (TMS) (Sigma-Aldrich, Munich, Germany) as internal standard, while coupling constants (*J*) were measured in Hz. Column chromatography was carried out on silica gel 230–400 mesh, Merck, (Merck, Bielefeld, Germany) and silica gel 70–230 mesh (Merck). Thin layer chromatography (TLC) was performed on Merck precoated silica gel 60 F_254_ aluminum foil (Merck), and spots were detected using ceric sulfate spray reagent after heating. The degree of purity of the positive control compounds was ≥98%, while that of the isolated compound was >95%. The molecular composition of the isolated compounds was identified by exact mass determinations. Gentamycin was purchased from Jinling Pharmaceutic (Group) Corp. All reagents used were of analytical grade.

### 3.2. Plant Material

Leaves and stem of the species *Vepris lecomteana* Pierre were collected in May 2016 close to the falls of Kampo (Kribi, South region of Cameroon) and identified by the botanist Nana Victor of the Herbier National du Cameroun, where voucher samples (Ref. 46574HNC) were deposited.

### 3.3. Extraction and Isolation

The air dried and powdered leaves (335.0 g) of *Vepris lecomteana* were extracted with methanol at room temperature for 72 h. After filtration and evaporation under reduced pressure at 40 °C, 24.8 g of dried crude extract were obtained and fractionated using vacuum liquid chromatography (VLC) with a mixture of petrol ether, ethyl acetate, and methanol vacuum liquid chromatography on the basis of TLC analysis, to afford fractions A (1.1 g) (20% of petrol ether in EtOAc), B (5.7 g) (50% of petrol ether in EtOAc), and C (8.5 g) (70% of petrol ether in EtOAc and 100% EtOAc). Fractions were subjected to column chromatography over silica gel 60 C (0.04–0.063 mm), and eluted with petrol ether followed by a mixture of petrol ether/EtOAc, using gradients of increasing polarity, and finally by EtOAc. 

Fraction A (1.1 g) was subjected to silica gel 60 C column chromatography eluted with petrol ether/EtOAc gradient to yield lupeol (17.5 mg) from combined fractions 1–6, and a mixture of *β*-sitosterol and stigmasterol (15.5 mg) together with myristyl alcohol (4.8 mg) from combined fractions 8–16. Fraction B was submitted to thin layer chromatography (TLC) for anisaldehyde/sulfuric acid spray reagent reaction, and not further followed up, due to lack of promising zones. Fraction C (5.7 g) was also chromatographed over silica gel column to yield lecomtequinoline A (**1**) (81.1 mg), lecomtequinoline B (**2**) (7.4 mg), and anhydroevoxine (**4**) (118.5 mg).

The air-dried and powdered stem (2.3 kg) of the plant was likewise extracted with methanol at room temperature for 72 h. After filtration and evaporation under reduced pressure at 40 °C, 45.2 g of dried crude extract were obtained, and fractionated with a mixture of petrol ether, ethyl acetate, and methanol, using VLC on the basis of TLC analysis, to afford fractions D (10.5 g) (20% of petrol ether in EA) and E (27.6 g) (70% of petrol ether in EA and 100% EA). The fractions were subjected to column chromatography over silica gel 60 C (0.04–0.063 mm), and eluted with petrol ether followed by a mixture of petrol ether/EtOAc using gradients of increasing polarity, and finally, by EtOAc. During the extraction of the stem powder, a white solid precipitated. After filtration and recrystallization using a mixture of petrol ether/EtOAc (1/3), hesperidin (401.5 mg) was obtained. Fraction D (10.5 g) was also treated—using the same approach as applied for fraction A—to receive 62 subfractions of around 100 mL each, which were collected and combined on the basis of TLC analysis. Combined subfractions 1–21 yielded lecomtequinoline C (**3**) (6.7 mg), and combined subfractions 23–30 afforded evoxine (**5**) (228.6 mg). Subfractions 25–27, 32–35, 48–50 gave a precipitate to afford lupeol (398.5 mg), a mixture of *β*-sitosterol and stigmasterol (14.4 mg), and dictamnine (**6**) (13.7 mg). Subfractions 55–62 were combined and subjected to silica gel 60 H column chromatography with petrol ether/acetone 85:15, to yield *N*-methylflindersine (**7**) (5.1 mg). 

Fraction E (27.6 g) was chromatographed over silica gel 60 C on a column with a petrol ether/EtOAc and EtOAc/MeOH gradient. A total of 28 fractions of around 100 mL each were collected and combined on the basis of TLC as well. The combined fractions 1–18 were further chromatographed over a silica gel 60 H column with petrol ether/EtOAc, to yield white fibers identified as evoxathine (**8**) (74.0 mg) and a white powder identified as *β*-sitosterol-3-*O*-*β*-d-glucopyranoside (20.0 mg).

### 3.4. Lecomtequinoline A *(****1****)*

White needles (CHCl_3_); m.p. 125–127 °C; *R_f_* = 0.44, silica gel 60 F_254_, hexanes/EtOAc (4/1); UV (MeOH) *λ*_max_ (log *ε*) 229 (3.20), 250 (4.40), 307 (4.19), 316 (4.14), 339 (4.10), 364 (4.14) nm; IR (KBr) *ν*_max_ 3180, 3030, 2974, 2929, 1616, 1574, 1363 cm^−1^; ^1^H and ^13^C-NMR data, see Table 1; HR-ESI-MS [M + Na]^+^ *m*/*z* 390.1730 (calcd.. for C_22_H_25_NO_4_Na, 390.1783).

### 3.5. Lecomtequinoline B *(****2****)*

White needles (CHCl_3_); m.p. 116–118 °C; *R_f_* = 0.40, silica gel 60 F_254_, hexanes/EtOAc (4/1); ${\left[\alpha \right]}_{D}^{25}$ −45.7 (*c* 0.08, MeOH); CD [MeOH, nm (Δ*ε*)] 250 (+4.82), 242 (+0.70); UV (MeOH) *λ*_max_ (log ε) 228 (3.10), 252 (4.30), 310 (4.20), 316 (4.10), 340 (4.15), 365 (4.16) nm; IR (KBr) *ν*_max_ 3118, 2969, 2860, 1674, 1576, 1360, 1290 cm^−1^; ^1^H and ^13^C-NMR data, see Table 1; ESI-MS (%) *m*/*z* 383.3 (C_22_H_25_NO_5__,_ 8), 346 (20), 315 (33), 231 (100), 230 (25), 159 (52), 129 (8); HR-ESI-MS [M]^+^ *m*/*z* 383.1728 (calcd.. for C_22_H_25_NO_5_, 383.1732).

### 3.6. Lecomtequinoline C *(****3****)*

White needles (CHCl_3_); m.p. 274–276 °C; *R_f_* = 0.41, silica gel 60 F_254_, hexanes/EtOAc (2/3); ${\left[\alpha \right]}_{D}^{25}$ −25.5 (*c* 0.10, MeOH); CD [MeOH, nm (Δ*ε*)] 276 (Δ*ε* +4.92), 232 (Δ*ε* +0.90); UV (MeOH) *λ*_max_ (log ε) 232 (3.15), 255(4.23), 305 (4.15), 325 (4.45), 360 (4.50), 370 (4.30) nm; IR (KBr) *ν*_max_ 3224, 3126, 2971, 1625, 1584, 1378, 1240 cm^−1^; ^1^H and ^13^C-NMR data, see Table 1; ESI-MS (%) *m*/*z* 315.3 (C_17_H_17_NO_5_, 15), 299 (98), 265 (54), 254 (50), 240 (100), 216 (14), 181 (84), 149 (45); HR-ESI-MS [M]^+^ *m/z* 315.1087, (calcd.. for C_17_H_17_NO_5_, 315.1106).

### 3.7. Antimicrobial Activities

The minimum inhibition concentrations (MICs) of test samples and the positive control drug gentamycin were measured by the microdilution broth susceptibility assay [24] against the bacteria *Escherichia coli* (DSMZ 1058), *Bacillus subtilis* (DSMZ 704), *Pseudomonas agarici* (DSMZ 11810), *Micrococcus luteus* (DSMZ 1605), and *Staphylococcus warneri* (DSMZ 20036)*,* obtained from DSMZ, Germany. The inocula of bacterial strains were prepared from 12 h broth cultures, and suspensions were adjusted to 0.5 McFarland standard turbidity. The samples were dissolved in 10% DMSO and diluted twofold in sterile 96-well microtiter plates, in duplicate, using BHI broth. Standardized inocula of test strains were added, and after incubation at 37 °C for 24 h on a rotary shaker at 200 rpm, MICs were read as the lowest concentration with inhibition of the growth of the test organisms, compared to the positive control gentamycin and medium containing 10% DMSO as negative control.

## 4. Conclusions

To the best of our knowledge, this is the first study on phytochemical and pharmacological properties of *Vepris lecomteana*. We report here, the isolation and structural elucidation of new furoquinoline alkaloids named lecomtequinoline A (**1**), B (**2**), and C (**3**), and their antibacterial activities, together with those of anhydroevoxine (**4**), evoxine (**5**), and dictamnine (**6**). The microdilution assay concerning antibacterial activity against *Escherichia coli*, *Bacillus subtilis*, *Pseudomonas agarici*, *Micrococcus luteus*, and *Staphylococcus warneri* resulted in MIC values displaying decreasing activities from crude extracts over fractions towards isolated compounds, suggesting synergistic effects of compounds, potentially involving **1**–**6**, as well as *N*-methylflindersine (**7**), evoxathine (**8**), lupeol, stigmasterol, *β*-sitosterol, *β*-sitosterol-3-*O*-*β*-d-glucopyranoside, and myristic alcohol. In accordance with other *Vepris* species, *Vepris lecomteana* might find applications in crude drug medicines, especially in Western African countries where the plant occurs endemically. Subject to determination of their in vivo toxicity profile, extracts of the leaves and stem might turn out to be valuable for treatment of bacterial infections caused by Gram-negative *Escherichia coli* responsible for certain forms of diarrhea; the Gram-positive *Bacillus subtilis* causing diarrhea, enteritis, and dermatosis*;* the Gram-positive *Micrococcus luteus* causing skin infections in immunosuppressed patients; as well as the Gram-positive *Staphylococcus warneri* suggested to be linked to spontaneous abortion, urinary tract infection, meningitis, and endocarditis.

## Figures and Tables

**Table 1:** ^1^H (500 MHz) and ^13^C (125 MHz) NMR assignments for (**1**–**3**) in CDCl_3._

Attribution	1		2		3	
	^1^H	^13^C	^1^H	^13^C	^1^H	^13^C
**2**	7.50 (d, *J* = 2.8)	143.0	7.51 (d, *J* = 2.8)	143.1	7.74 (d, *J* = 2.8)	142.8
**3**	6.96 (d, *J* = 2.8)	104.7	6.96 (d, *J* = 2.8)	104.6	7.26 (d, *J* = 2.8)	105.0
**3a**	-	101.9	-	102.2	-	101.9
**4**	-	157.1	-	157.1	-	157.7
**4a**	-	114.9	-	115.5	-	113.8
**5**	7.90 (d, *J* = 9.3)	117.5	7.91 (d, *J* = 9.3)	117.9	7.65 (d, *J* = 9.3)	114.3
**6**	7.13 (d, *J* = 9.3)	114.5	7.16 (d, *J* = 9.3)	114.9	7.00 (d, *J* = 9.3)	115.4
**7**	-	151.9	-	151.6		142.8
**8**	-	141.8	-	142.1	-	135.5
**8a**	-	142.1	-	142.1	-	136.9
**9a**	-	164.1	-	164.2	-	163.8
1′	4.75 (d, *J* = 7.1)	70.3	4.32 (dd, *J* = 11.3; 4.9) 4.21 (dd, *J* = 11.3; 5.6)	69.4	4.44 (dd, *J* = 11.1, 2.0) 4.04 (dd, *J* = 11.1, 9.3)	65.3
2′	5.67 (dd, *J* = 7.1, 5.8)	121.3	3.16 (dd, *J* = 5.6; 4.9)	61.6	3.95 (dd, *J* = 9.3, 2.0)	61.8
3′	-	137.4	-	58.3	-	58.7
4′	1.66 (s)	25.8	1.32 (d, *J* = 1.5)	24.6	1.37 (d, *J* = 1.5)	25.5
5′	1.59 (s)	18.0	1.28 (d, *J* = 1.5)	18.9	1.36 (d, *J* = 1.5)	23.5
1″	4.68 (d, *J* = 6.7)	66.9	4.77 (d, *J* = 7.1)	70.5	-	-
2″	5.49 (dd, *J* = 8.2, 5.2)	120.3	5.67 (dd, *J* = 7.2, 5.8)	121.1	-	-
3″	-	137.6	-	137.7	-	-
4″	1.71 (s)	25.8	1.67 (s)	25.8	-	-
5″	1.69 (s)	18.3	1.61 (s)	18.1	-	-
OH	-	-	-	-	4.55 (brs)	-
CH_3_O	4.35 (s)	58.9	4.35 (s)	59.0	4.43 (s)	58.6

Assignments were based on HMQC, HMBC, and NOESY experiments.

**Table 2:** Minimum inhibition concentration (MIC, μg/mL) of leaf and stem extracts, fractions and compounds (**1**–**6**) from *Vepris lecomteana*.

Specimen	Microorganism				
	*E. coli*	*B. subtilis*	*P. agarici*	*M. luteus*	*S. warneri*
Leaf Extract	13.2	10.1	10.5	12.4	16.5
Stem Extract	14.3	11.0	10.2	13.8	20.5
Fraction A	18.7	16.7	15.5	not active	not active
Fraction B	10.5	11.5	10.5	10.7	13.5
Fraction A′	18.2	18.5	not active	not active	19.2
Fraction B′	10.5	12.0	10.8	10.9	15.5
Fraction C′	11.7	11.2	10.1	4.5	10.4
**1**	18.7	11.1	16.2	12.0	not active
**2**	16.2	not active	16.9	12.9	not active
**3**	15.7	12.5	15.9	12.3	not active
**4**	not active	15.3	16.5	not active	not active
**5**	not active	not active	17.0	not active	not active
**6**	not active	17.7	15.1	not active	not active
Gentamycin	1.0	1.9	1.0	0.2	1.0

## Supplementary Materials

The spectra of compounds (**1**–**3**) are available online.
